# DNA methylation, microRNA expression profiles and their relationships with transcriptome in grass-fed and grain-fed Angus cattle rumen tissue

**DOI:** 10.1371/journal.pone.0214559

**Published:** 2019-10-17

**Authors:** Yaokun Li, José A. Carrillo, Yi Ding, Yanghua He, Chunping Zhao, Jianan Liu, Linsen Zan, Jiuzhou Song

**Affiliations:** 1 College of Animal Science, South China Agricultural University, Guangzhou, Guangdong, P.R. China; 2 Department of Animal & Avian Sciences, University of Maryland, College Park, Maryland, United States of America; 3 College of Animal Science and Technology, Northwest A&F University, Yangling, Shaanxi, P.R. China; University of Florida, UNITED STATES

## Abstract

Rumen is an organ for supplying nutrients for the growth and production of bovine, which might function differently under grass-fed and grain-fed regimens considering the association of gene expression, DNA methylation, and microRNA expression. The objective of this study was to explore the potential mechanism influencing rumen function of grass-fed and grain-fed animals. Methylated DNA binding domain sequencing (MBD-Seq) and microRNA-Seq were respectively utilized to detect the DNA methylation and microRNA expression in rumen tissue of grass-fed and grain-fed Angus cattle. Combined analysis revealed that the expression of the differentially expressed genes ADAMTS3 and ENPP3 was correlated with the methylation abundance of the corresponding differentially methylated regions (DMRs) inside these two genes, and these two genes were reported to be respectively involved in biosynthesis and regulation of glycosyltransferase activity; the differentially expressed microRNA bta-mir-122 was predicted to possibly target the differentially expressed genes OCLN and RBM47, potentially affecting the rumen function; the microRNA bta-mir-655 was exclusively detected in grain-fed group; its targets were significantly enriched in insulin and TGF-beta signaling pathways, which might worked together to regulate the function of rumen, resulting in different characteristics between grass-fed and grain-fed cattle. Collectively, our results provided insights into understanding the mechanisms determining rumen function and unraveled the biological basis underlying the economic traits to improve the productivity of animals.

## Introduction

Historically, most of beef products were from grass-finished cattle. Since the 1950’s, numerous studies were performed to improve the efficiency of beef production; meanwhile, the beef cattle feedlot industry began to emerge, where high-energy grains were utilized to improve the productivity of cattle. And, consumers have been accustomed to grain-fed beef, considering the flavor and palatability resulted from the diets with large proportion of high-energy grain [1]. However, due to new research on the effects of diverse feeding regimens, consumers’ preferences for beef quality have changed, making certain producers to regress to the pastoral beef production in spite of the feeding inefficiency. Recently, growing number of consumers are interested in products obtained from grass-finished animals, raising concerns about the quality difference between grass-fed and grain-fed beef [2]. During the past few decades, it was elucidated that altered feeding regimes could bring about changes in the nutritional quality of beef. The essential fatty acids omega-3 and omega-6 are critical for animal health; however, they cannot be produced by the host and must be obtained from food [2]. Studies illustrated that grass-fed beef had a significantly higher content of omega-3 and displayed a more favorable omega-3/omega-6 ratio compared with grain-fed beef [3–4]. Beta-carotene could modulate immune reaction and prevent animals from bacterial and viral infection. Compared with grain-fed animals, beta-carotene was more abundant in the muscle of grass-fed animals [5–8]. Diterpenoids and derivatives of chlorophyll were also higher in grass-fed beef than in grain-fed beef, which played important roles in changing the flavor and aroma of the cooked beef [9]. Additionally, higher level of vitamin E was found in the grass-fed beef than the beef products from concentrate diets [10]. Besides nutritional components, the transcriptomic difference of cattle could also be detected due to varied feeding regimens [11–12].

In the field of genetics, epigenetics refers to cellular and physiological phenotypic trait alterations because of external factors that can influence genes expression without changing the DNA sequence [13]. As one important type of the epigenetic mechanism, DNA methylation was generally used to regulate gene expression; disturbing it could cause abnormal expression of certain genes [14]. DNA methylation has been largely studied due to its involvement in regulating most of the biological processes, including embryonic development, chromosome stability, chromatin structure, and transcription [15–19]. DNA methylation can interfere with transcription factor binding, indirectly repressing the gene activity through the recruitment of methyl-CpG binding domain (MBD) protein that can alter chromatin structure [20]. The repression strength could be determined by the concentration of DNA methylation. In porcine, differentially methylated regions in the promoter have been found to be associated with the repression of genes related to obesity and some novel genes [21]. Up to now, genome-wide DNA methylation profiles in many animal breeds, including chicken, pig, arabidopsis, human and bovine, have been reported [21–26]. However, the DNA methylation pattern of bovine rumen tissue was rarely explored.

Additionally, the gene expression could also be regulated by miRNAs, which could specifically bind to corresponding mRNAs, leading to the mRNA silencing and translation repression. It is known that each miRNA might have many mRNA targets; and each mRNA could also be regulated by more than one miRNA [27]. In mammals, miRNAs are predicted to regulate the expression of ~60% of all protein-coding genes, and found to be associated with many biological processes, including cell apoptosis, cell growth and differentiation, and embryo development [28–29].

As the largest part of the stomach, rumen constitutes the important workplace for feed digestion and microorganism fermentation, which is critical for supplying nutrients for animals’ growth, development and production. Given the emerging roles of miRNAs and DNA methylation in gene regulation, identifying the DNA methylation profile and expression pattern of miRNAs is critical to understand their functions in the process of bovine development. Therefore, we utilized Illumina sequencing technology to explore the miRNA expression pattern and DNA methylation profile in the rumen tissue of Angus cattle raised under grass-fed and grain-fed regimens. Further, we conducted the combined analysis of miRNAs, DNA methylation, and mRNA expression, which was important for further functional study and valuable biomarkers detection. Accordingly, our results would be significant for exploring potential mechanisms that led to differences observed between animals under altered feeding regimens.

## Materials and methods

### Ethics statement

We conducted the animal experiments according to the NIH guidelines for housing and care of laboratory animals. The Institutional Animal Care and Use Committee (IACUC) of University of Maryland at College Park reviewed and authorized the protocols (permit number R-08-62).

### Sample collection

The studied Angus cattle were born and raised at the Wye Angus farm. This farm has produced many progenies with similar genetic background. All experimental animals in this study received the same diet until weaning. After weaning, the calves were randomly assigned to one diet (grass-fed or grain-fed) and followed that regimen until slaughter. The grain-fed steers received conventional diet comprised of trace minerals, shelled corn, corn silage and soybean. The grass-fed steers normally ate grazed alfalfa; in winter, we provided bailage for the animals. The grass-fed animals consumed no industrial or agricultural byproducts and did not receive any type of grain. The grain-fed individuals met the market weight around the age of 14 months; grass-fed steers reached the similar weight value with the age of 20 months. After slaughter at the Old Line Custom Meat Company (Baltimore, MD), the rumen samples from all experimental animals were immediately excised at the same location around the cardiac ostium, and then the samples were rinsed and kept at -80°C for subsequent analysis. For MBD-Seq, two animals were contained per experimental group. Based on the MBD-Seq experiment, we respectively added one animal per group in miRNA-Seq; thus, three animals were respectively utilized in grass-fed and grain-fed group for miRNA-Seq analysis.

### DNA extraction and MBD-Seq library construction

Firstly, we extracted the genomic DNA from the four experimental animals through the Wizard Genomic DNA purification kit (Promega, A1120), and measured the concentration via the Qubit dsDNA Broad-Range Assay (Invitrogen, Q32850). The genomic DNA from each individual was adjusted to 0.1μg/μl for a final volume of 55 μl and sheared into 300–500 bp fragments. Secondly, MethylCap kit (Diagenode, C02020010) was employed to obtain the methylated DNA (meDNA). The 141.8 μl of capture reaction mix containing 12 μl of sheared DNA was prepared according to the instruction of the kit. Then, the capture reaction mix (119 μl) was incubated with 1 μl of diluted MethylCap protein at 40 rpm for 2 hours at 4°C. The rest 22.8 μl was utilized as input sample. Next, meDNA Capture beads (coated with GSH) provided by the kit were utilized to capture methylated DNA. The bead pellet combined by the methylated DNA was collected. After washing the bead pellet, we performed the elution of the captured DNA; 150 μl of low, medium, and high concentration elution buffer were sequentially utilized per reaction. All fractions were treated by the MiniElute PCR Purification Kit (QIAGEN, 28006).

We repaired the end of the fragmented methylated DNA by using the NEBNext End Repair Module (NEB, E6050S, USA). Then the 3’ poly “A” was added through DNA Polymerase I, Large (Klenow) Fragment (NEB, M0210L, USA). Next, we ligated a pair of Solexa adaptors (Illumina) to the repaired ends by T4 ligase (Promega, M1801, USA). The fragments of the ligated products (DNA plus adaptors) from 200 to 500bp were selected from 2% agarose gels and purified by QIA- quick Gel Extraction Kit (QIAGEN, USA). Then, the Phusion Hot Start High-Fidelity DNA Polymerase (NEB, M0530S, USA) was used to enrich the purified DNA templates through PCR. After purification of the PCR products (MinElute PCR Purification Kit, QIAGEN, USA), the concentration of the DNA library was measured through the Qubit assay (Life Technology, Q32850). Finally, the DNA sequencing was performed in the Solexa 1G Genome Analyzer (Illumina) according to the specification provided by the manufacturer.

### MBD-Seq data analysis

We evaluated the quality of the raw reads through FastQC, which is a web-based software. It could thoroughly examine the reads and create a detailed quality assurance report containing “per base sequence quality” and “per sequence GC content”, etc. Then, Bowtie was used to align the sequencing reads to the reference genome (Bos_taurus_UMD3.1/bosTau6) [30], which was obtained from the iGenomes web site (http://support.illumina.com/sequencing/sequencing_software/igenome.html). During this step, according to the indicators generated by FastQC, we trimmed the first 10 bases and the last 5 bases of each read (50 bp) to keep high sequence quality score; and 35 bp tags were then obtained. For data format conversion, SAMtools and BEDtools were applied in our analysis.

For peaks identification, the Model Based Analysis of ChIP-Seq (MACS) was employed [31]. Identification of the DMRs was performed by the R package Diffbind; it calculates the differentially bound sites using affinity data. The input data for DiffBind was the bam file containing the aligned reads and the peaks set identified by MACS. The default method TMM (Trimmed Mean of M-values) considering the effective library size was used for normalization. After creating a contrast between conditions, DiffBind carried out a DESeq2 analysis with a false discovery rate (FDR) < 0.1 to call the DMRs. DESeq2 approach employed shrinkage estimation for fold changes and dispersions, making the estimated values more stable and interpretable [32].

The ChIPpeakAnno package was utilized for the genomic annotation of the previously identified DMRs [33]. This software provides DMRs information about the distance, relative position and overlaps for the inquired features, including the information relative to the exon and the transcriptional start site (TSS) of the corresponding genes. The annotation information was obtained from BioMart, using Ensembl 80 in the archive site; dataset “Bos taurus_genes_ensembl (UMD3.1)” corresponding to the bosTau6 reference genome was used for alignment. The CpG islands annotation was retrieved from the UCSC web browser. Finally, the DMRs were annotated based on specific genomic features. In addition, we combined the annotation results of DMRs and differentially expressed genes (DEGs) which has been published in 2015 [34], and performed the association analysis between the methylation abundance of the DMRs and the expression level of their corresponding genes including the DEGs between grass-fed and grain-fed Angus cattle through software SPSS Statistics 17.0, from which we hypothesized the relationship between the methylation abundance of the DMRs and the expression level of their corresponding genes.

### Bisulfite sequencing for MBD-Seq Validation

After quality evaluation and quantification, equal amounts of genomic DNA from two experimental animals of each group were pooled together, which were then served as the template for the bisulfite conversion. Firstly, 500 ng of each DNA pool was treated by the sodium bisulfite conversion reagents (Methyl EdgeTM Bisulfite Conversion System, Promega, USA). We randomly selected the DMRs from the bioinformatics analysis results for validation. The PCR primers were designed via MethPrimer (http://www.urogene.org/methprimer/) and shown in S1 Table. Then, PCR products were purified using QIA- quick Gel Extraction Kit (QIAGEN, USA), which were subsequently ligated to pGEM-T Vector (pGEM-T Vector System I, Promega, USA), and transformed to DH5α competent cells (Z-Competent E. Coli Cells—Strain Zymo 5α, ZYMO Research, USA) for screening successful insertions (blue-white selection). Next, ten white colonies from each culture plate were cultured overnight at 37°C in a shaker. The plasmid DNA was isolated utilizing Zyppy Plasmid Miniprep Kit (ZYMO Research, USA). BigDye Terminator v3.1 Cycle Sequencing Kit (Applied Biosystems, 4337456) was used for the sequencing in the ABI 3730 machine. Bisulfite sequencing results were analyzed through QUMA (http://quma.cdb.riken.jp).

### RNA extraction and microRNA-Seq library construction

We extracted the total RNA from the six experimental individuals (three animals per group) by Trizol (Invitrogen, Carlsbad, CA, USA), the RNA was then treated by DNase digestion and purified by Qiagen RNeasy column (Invitrogen, Carlsbad, CA, USA), as previously described [35]. We checked the integrity and quality by a NanoDrop 1000 spectrophotometer and by visualization on a 1.5% agarose gel. The microRNA-Seq library was built utilizing NEBNext Multiplex Small RNA Library Prep Set for Illumina (Set 1) following the instructions provided by the manufacturer (NEB, E7300S/L, USA). The library construction was started with 800ng total RNA. Firstly, the 3’ adaptor and 5’ adaptor were sequentially ligated to the RNA. Then, we performed the reverse transcription to synthesize the first strand. Next, we executed the PCR amplification and added the 6-bp index to the DNA products; different libraries were assigned different indexes. Subsequently, we selected and recycled the DNA fragments from 140 to 150 bp using 6% polyacrylamide gel (6% Novex^®^ TBE PAGE gel, Life Technology, USA), which were corresponding to RNAs from 21 to 30 bp. After purification, the concentration was measured by the Qubit assay (Life Technology, Q32850). Finally, we sequenced the libraries identified by the 6-bp index through an Illumina HiSeq 2000 sequencer, as described previously [36].

### microRNA-Seq data analysis

After quality evaluation of the tags performed by FastQC, all samples were analyzed employing miRDeep* software, which could quantify known and novel miRNAs [37]. This program had a user-friendly graphic interface implemented totally in java and accepted raw data in FastQ and SAM/BAM format as input. The default length of miRNAs was set as 18–23 nucleotides. Low-quality reads were filtered out at the alignment stage; the read with a Phred quality score of 20 or higher was considered as good read. Meanwhile, multi-mapping reads with alignments to more than 100 genomic loci were also filtered out; the score based on the probabilistic score of the potential miRNA precursor was set as -10 as default [37]. In the further miRNA expression analysis, we only considered known miRNAs. Expression values for known miRNAs were estimated individually by miRDeep* in each sample. Subsequently, these expression levels were extracted and recorded in a matrix, which was used later as input for the edgeR package to call the differentially expressed miRNAs.

After identification of the differentially expressed miRNAs, the target genes of those miRNAs were predicted by TargetScan [38], which were then utilized for the combined analysis with transcriptome analysis results (including the DEGs) published in 2015 [34]. We screened the overlapped genes of the differentially expressed miRNAs targets and the DEGs; based on the discovered overlapped DEGs, we then searched the corresponding miRNAs and performed the association analysis between the expression of the differentially expressed miRNAs and the corresponding DEGs through software SPSS Statistics 17.0, from which the relationship between the differentially expressed miRNAs and the DEGs could be analyzed.

### Quantitative real-time PCR (qRT-PCR) analysis

qRT-PCR was performed to verify the differentially expressed miRNAs found in the microRNA-Seq analysis on the iCycler iQ PCR system (Bio-Rad, Hercules, CA, USA). The qRT-PCR reactions were performed with a QuantiTect SYBR Green PCR Kit (Qiagen, Valencia, CA, USA) according to the manufacturer’s instructions. We conducted three technical replicates and two independent biological replicates for each product. RPS18 was chosen as the control gene [39]. The primer sequences were listed in S2 Table.

## Results

### Landscape of the DNA methylomes

**Fig 1:**
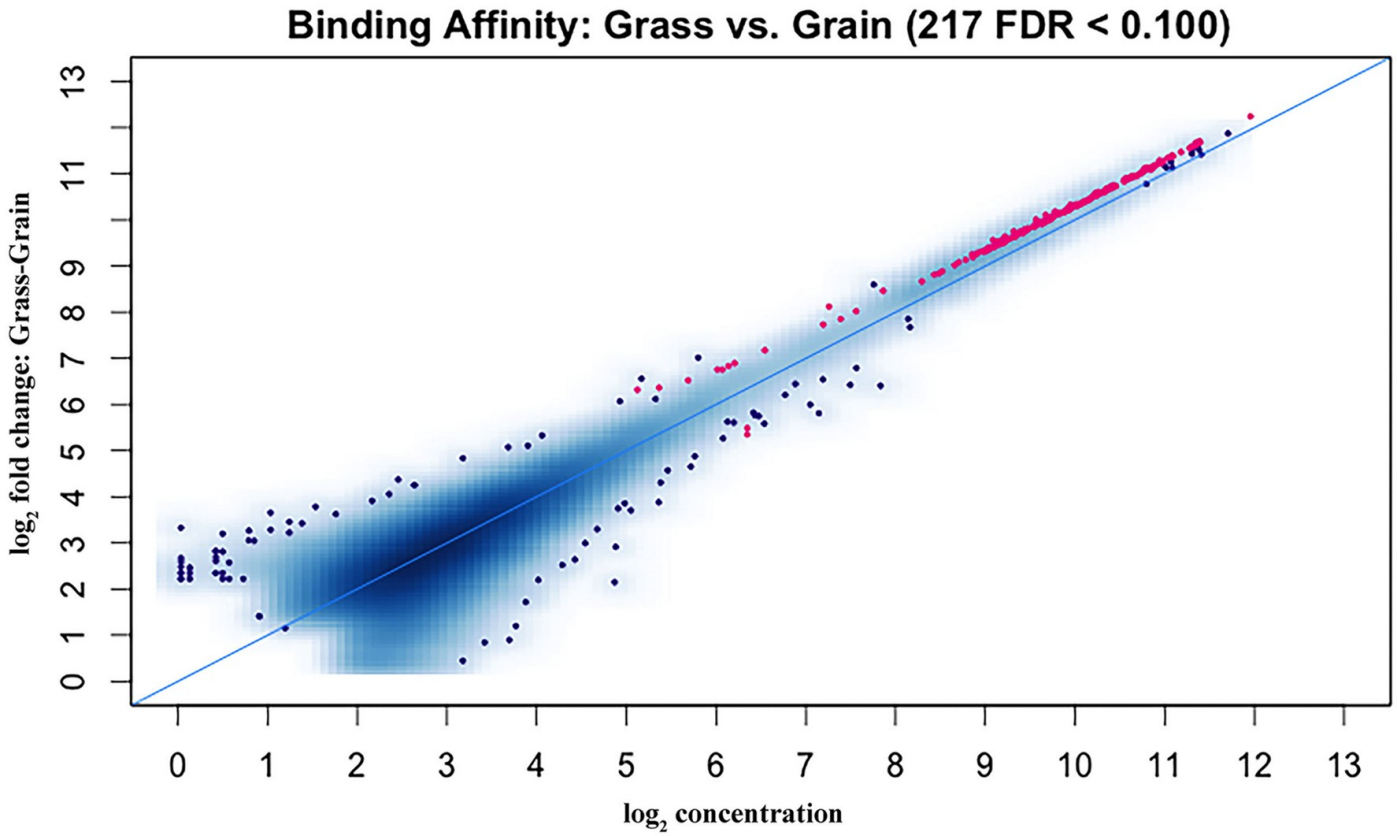
DMRs between grass-fed and grain-fed steers. The MA plot shows in red the DMRs obtained with a false discovery rate of < 0.1.

**Fig 2:**
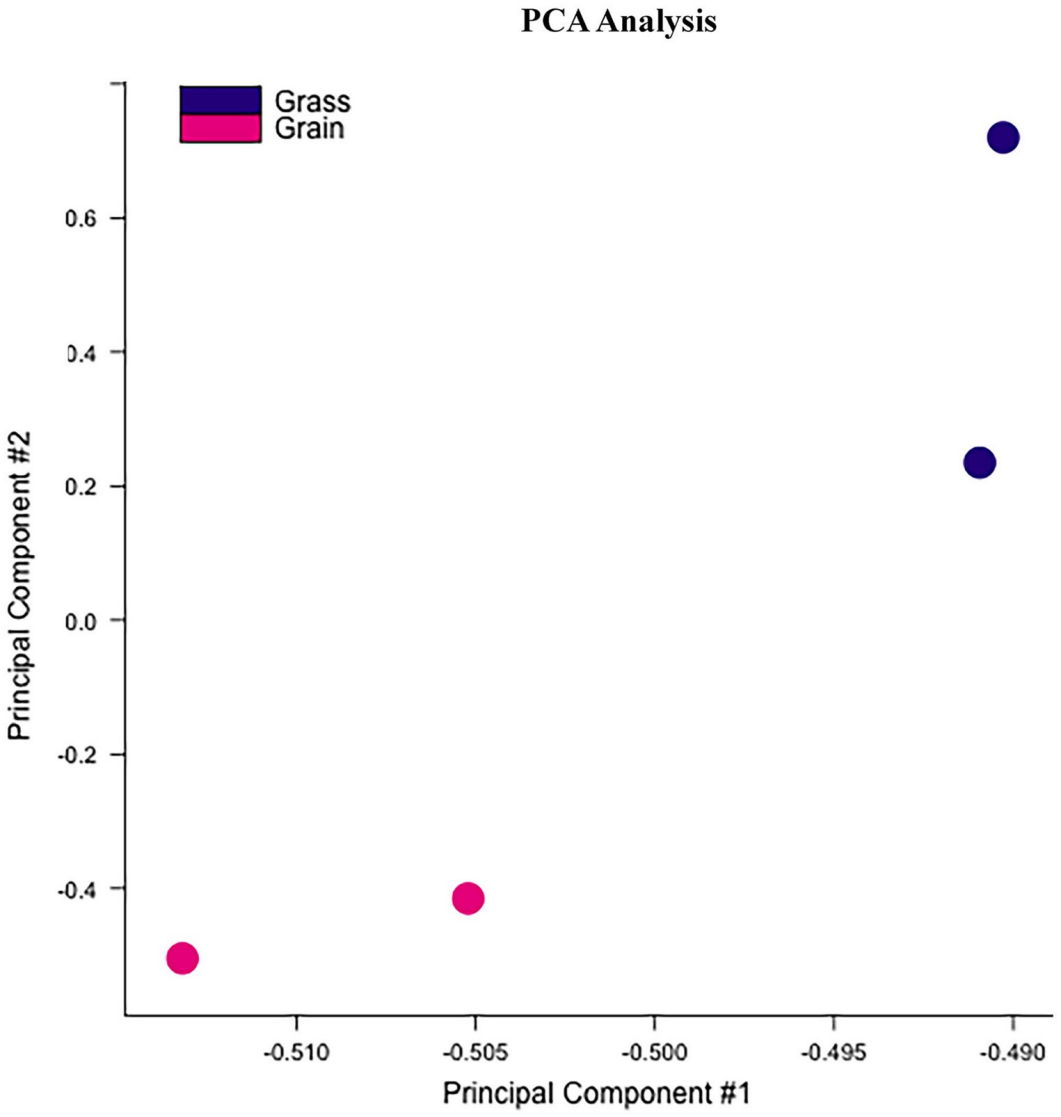
PCA analysis of the experimental individuals based on the identified DMRs. PCA: principle component analysis.

**Fig 3:**
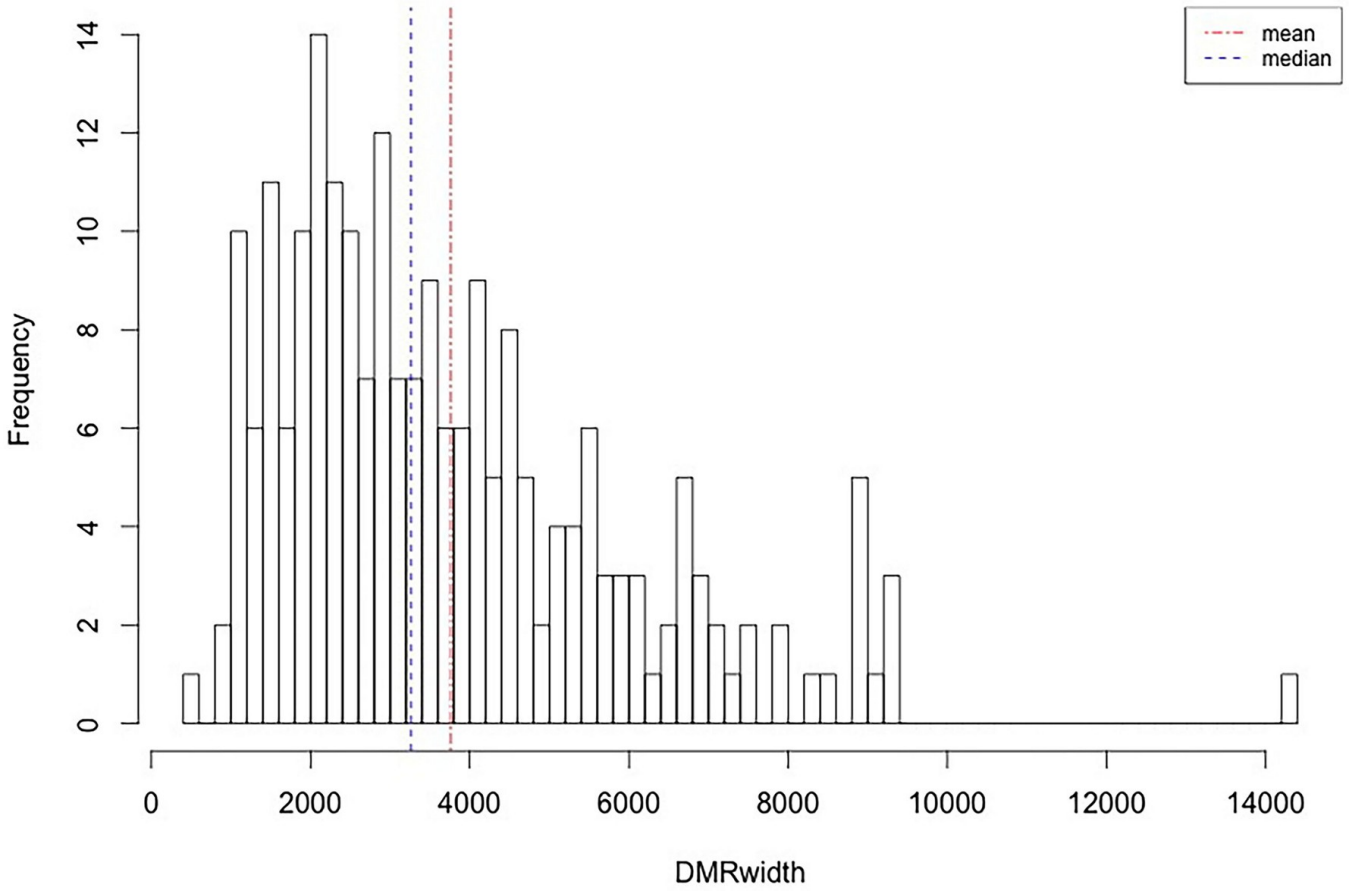
DMRs’ length density. The abscissa represents the extent of the DMRs in base pairs. The dotted light blue and red lines correspond for the median and mean respectively.

**Fig 4:**
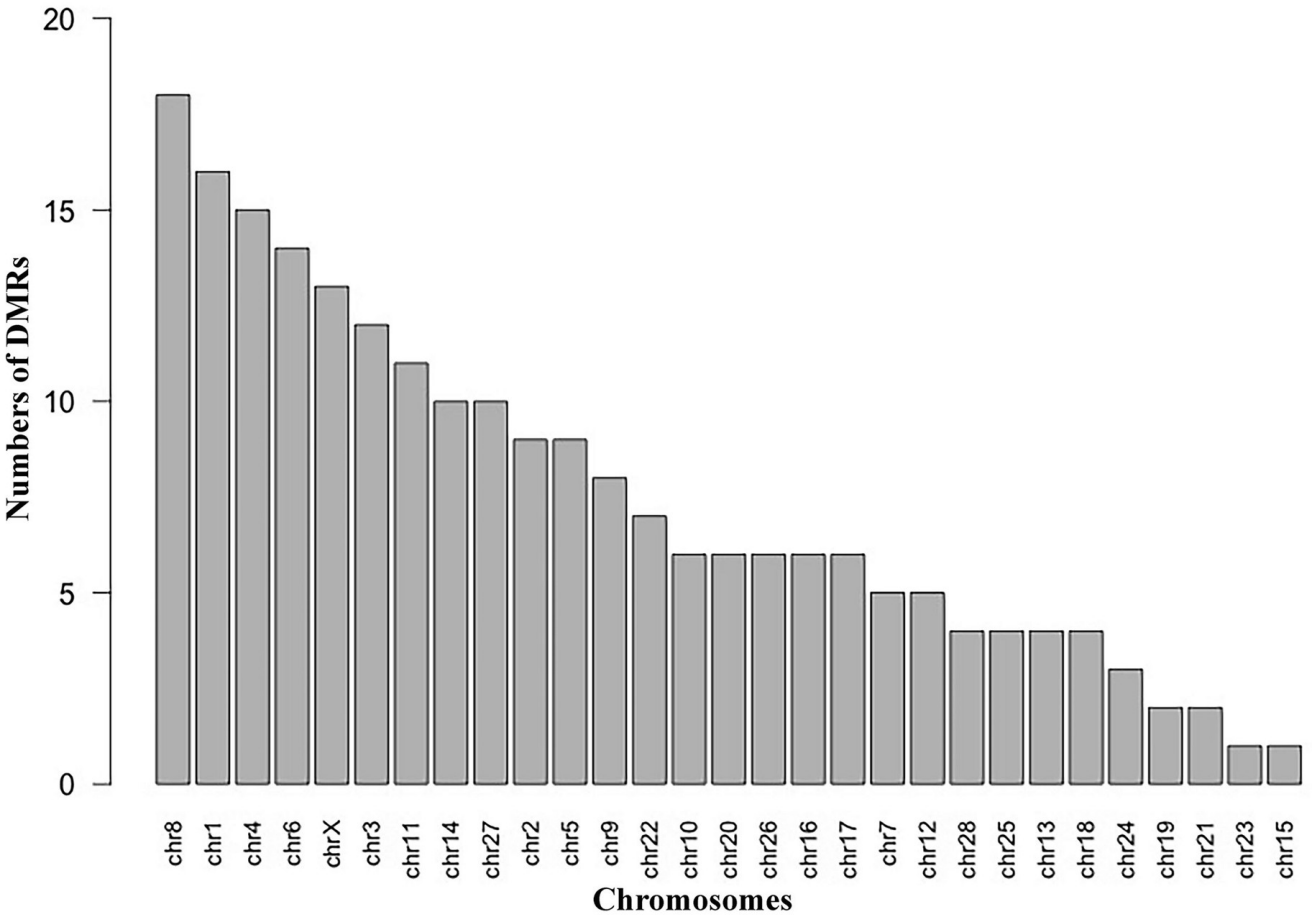
Chromosomal location frequencies of the DMRs. Distribution of the DMRs per chromosome without normalization (ignoring chromosome length).

For the methylome study, we had four experimental samples in total; the alignment levels were 94.92%, 82.98%, 95.54% and 96.10%, respectively (S1 Fig). We finally found 217 DMRs between the two groups (S3 Table), which were presented as red dots in Fig 1. Based on the identified DMRs, the cluster analysis of our experimental samples was performed (Fig 2), the results showed consistency with the group assignment of the samples, suggesting that these DNA fragments carrying DMRs could be used to predict the potential mechanism causing the difference between our two groups. From those DMRs, we found that only two DMRs were highly methylated and all the others low methylated in the grain-fed bovines compared with the grass-fed group. For the distribution of the DMR length, the average was 3760 bp with extreme value of 485 bp and 14,387 bp. Approximately one percent of the DMRs were less than 1,000 bp and 37.8% of the DMRs accounted for fragments longer than 4,000 bp (Fig 3). Fig 4 showed the number of DMRs per chromosome, chromosome 8 accounted for 18 DMRs indicating the largest number, following by chromosome 1 with 15 DMRs. Chromosomes 19, 21, 23, and 15 had the lowest number of DMRs with values of 2, 2, 1 and 1, respectively.

**Fig 5:**
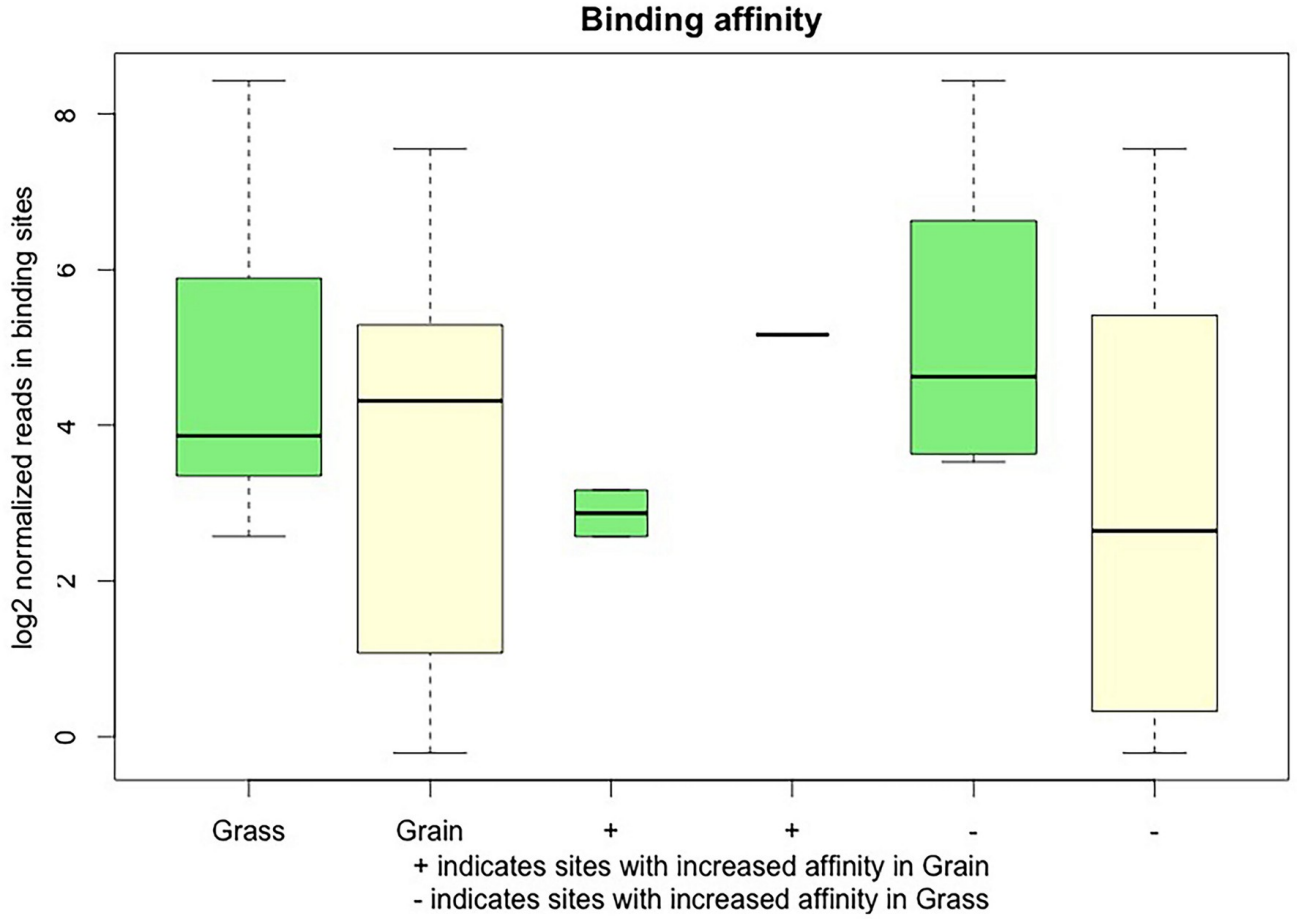
Normalized reads at the binding sites per condition. The first two boxplots represent the overall methylation level in the grass-fed and grain-fed groups. The + sign marked the sites with increased binding affinity in grass-fed group, the - sign represented the regions that enhanced the binding ability in the grain-fed group. The light green and yellow boxes corresponded to grass-fed and grain-fed with + and -, respectively.

**Fig 6:**
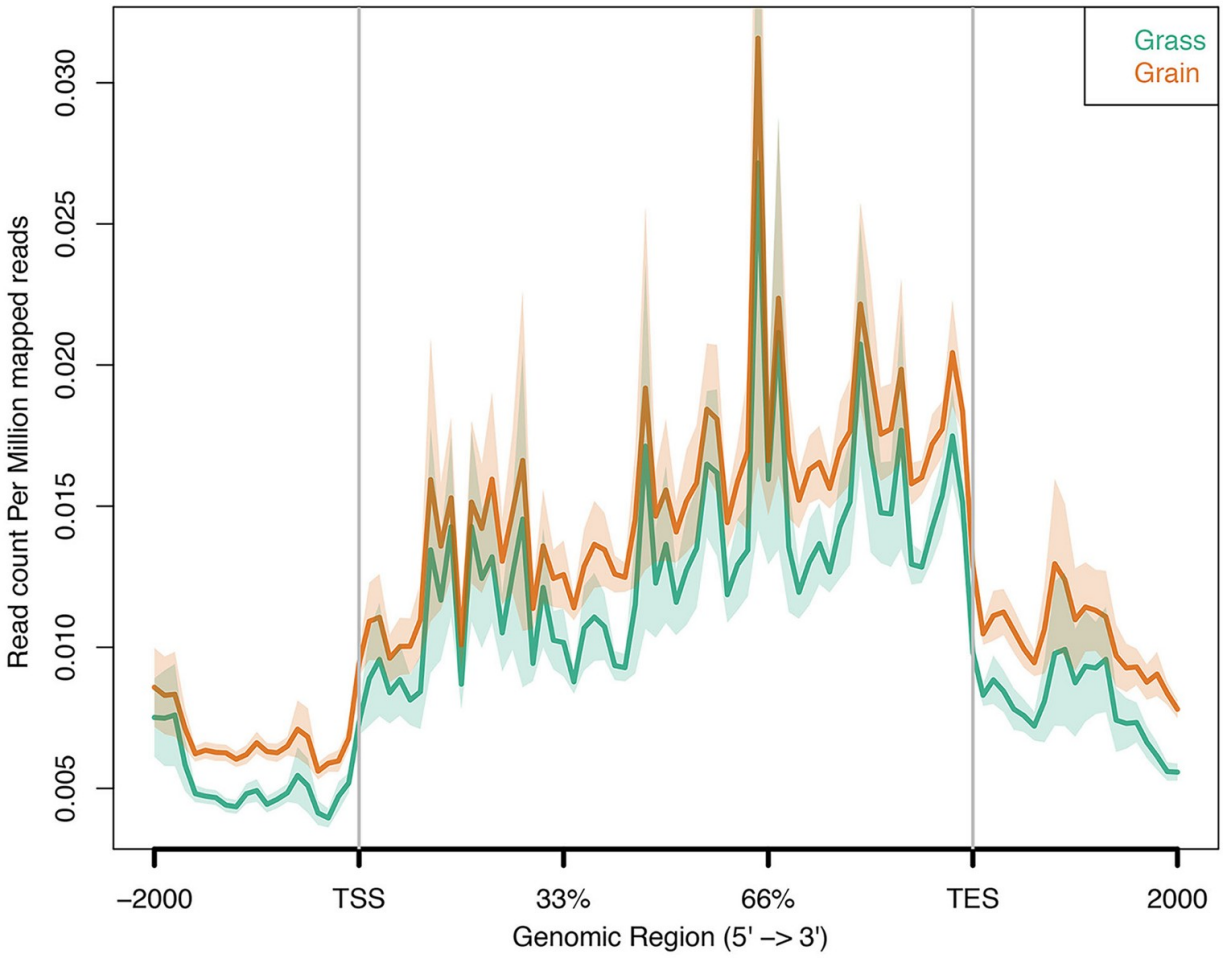
DNA methylation patterns around gene bodies in grass-fed and grain-fed animals measured by MBD-Seq. The x axis indicates the position around gene bodies, and the y axis indicates the normalized read number aligned to the normalized gene body region and the around region.

The binding affinity between grass-fed and grain-fed cattle was shown in Fig 5. We found that global DNA methylation level decreased in grain-fed individuals. Then, we detected the DNA methylation level in the 2 kb region upstream of the transcription start site (TSS), the 2 kb region downstream of the transcription end site (TES) and the gene body region from TSS to TES. In our experimental groups, the DNA methylation level declined significantly before the TSS and increased notably towards the gene body region with slight changes before the TES, followed by a sharp decrease in the downstream of TES (Fig 6). Compared with grass-fed individuals, the grain-fed steers showed a higher level of DNA methylation around the gene body region.

**Fig 7:**
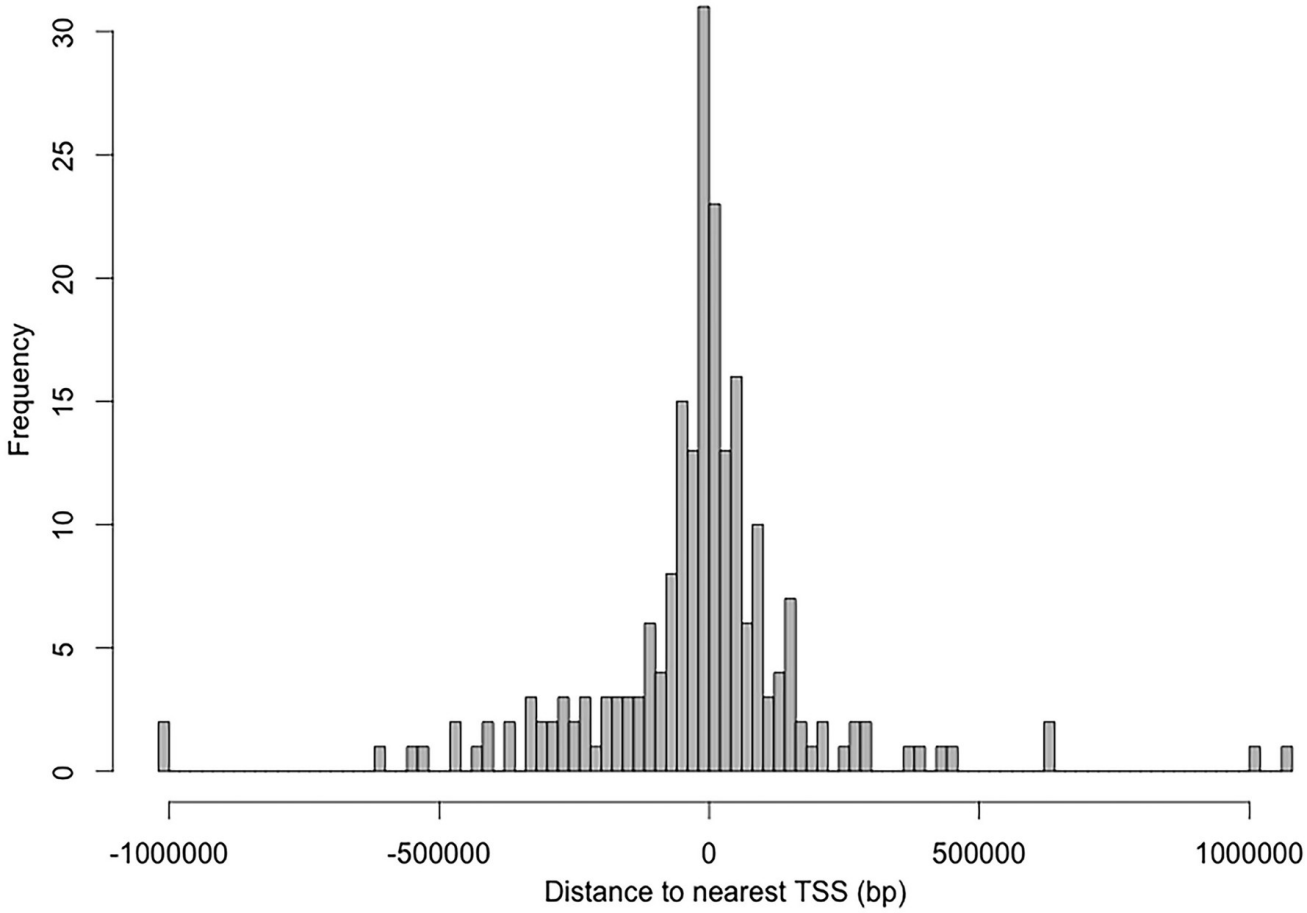
Frequency of DMRs’ distance to closest transcription start site. The distance from the transcription start site is represented in base pairs from the 0 in the x axis.

**Fig 8:**
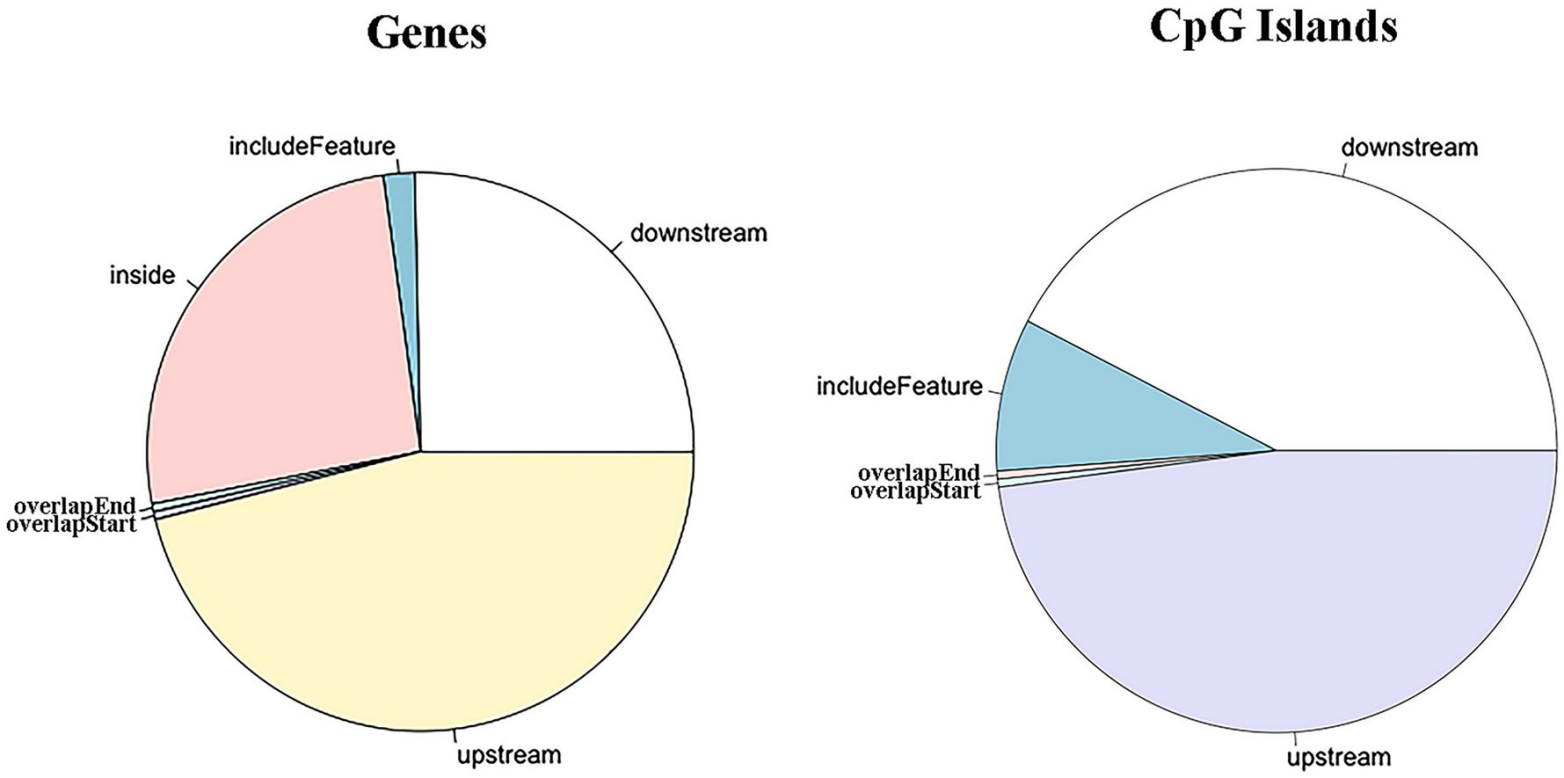
DMRs location distribution regarding genes and CpG islands. The labels in both pies represent: inside, DMR contained within the feature; include feature, the genomic feature is entirely included in the DMR; overlap start, the DMR extend over the start site of the feature; overlap end, the ending site of the feature overlaps with the DMR; downstream, DMR locates downstream the feature; upstream, the DMR aligns upstream the genomic feature.

The DMR annotation was subsequently performed (S4 Table). The distribution of DMRs’ distance to the nearest TSS could be found in Fig 7, suggesting that approximately 14.3% of the DMRs are located within a range of 10,000 bp from the TSS. Moreover, DMRs’ location related to genes and CpG islands were summarized in Fig 8. We found that almost a quarter of the DMRs were contained within genes and half in regions upstream of the TSS. The DMRs downstream of the genes accounted for 25%. Only four genes were found in the DMRs. Regarding CpG islands, approximately 8.8% of the annotated CpG islands were included in the DMRs. Most of the DMRs were outside of the CpG island boundaries with 42.4% upstream and 50% downstream of the CpG islands.

### Combined analysis of MBD-Seq with transcriptome (RNA-Seq)

In this study, we screened the genes with promoters containing DMRs, using 10,000 bp as the parameter for maximum distance. From the 217 identified DMRs, 21 were located within the promoters, corresponding to 21 different genes. Our previous study explored the transcriptome profiling in grass-fed and grain-fed animals, and 342 DEGs (FDR < 0.1) were detected between the two groups [34]. We found that, of the above 21 genes, gene SEC22C (Ensemble gene ID: ENSBTAG00000006570) was positively correlated with the corresponding DMR (r > 0.9, p = 0.055), gene HGSNAT (Ensemble gene ID: ENSBTAG00000033182) was negatively correlated with the corresponding DMR (r < -0.9, p = -0.029), however, none of the them could be found in the DEGs list; the p-value of the other genes were all larger than 0.1 (S5 Table). Additionally, 57 DMRs were found inside 52 genes, and all of the DMRs were highly methylated in grass-fed steers. And two of the corresponding genes were detected in the DEGs list which were ADAMTS3 and ENPP3, respectively; ADAMTS3 (Ensemble gene ID: ENSBTAG00000006507) was negatively correlated with the corresponding DMRs (r < -0.6), ENPP3 (Ensemble gene ID: ENSBTAG00000020196) was also negatively correlated with the DMR (r = -0.143), however, the p-value of these two genes was all larger that 0.05; generally, we found that 55.8% of the corresponding 52 genes were negatively correlated with the DMR, only one showed significant correlation (r = -0.967, p < 0.05) (S6 Table). Meanwhile, we found that 4 genes were inside the DMRs; however, none of them were differentially expressed. Based on the annotation regarding the TSS, another two genes could also be discovered in the DEGs list, which were CRISPLD1 and PRR5; CRISPLD1 was negatively correlated with the corresponding DMR and PRR5 was positively correlated with the DMR; however, the difference was not significant (p > 0.05); the nearest distance of the corresponding DMR to the TSS was respectively 14,973 and 45,887 bp.

### MBD-Seq and microRNA-Seq data validation

**Fig 9:**
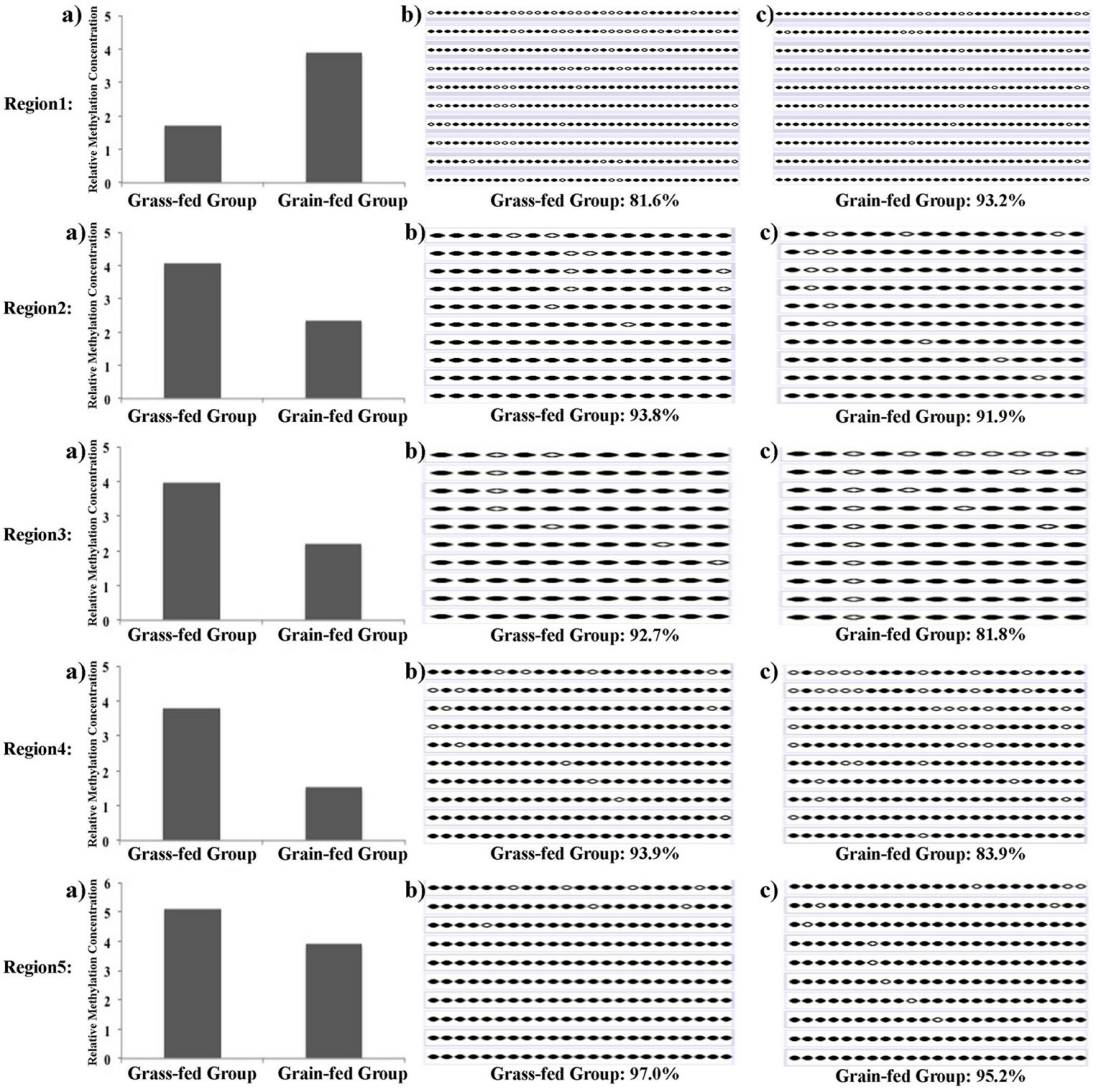
Bisulfite sequencing validation of MBD-Seq results. a) Methylation concentration levels from MBD-seq. b) and c) Bisulfite sequencing results. Each line represents a plasmid sequence and each dot indicates a CpG site. An open circle indicates an unmethylated CpG site and a black dot stands for one methylated CpG site. The methylation level was calculated as the number of methylated CpG sites divided by the total detected CpGs.

In order to assess the reliability and accuracy of DMRs detection from MBD-Seq, 5 regions were randomly selected for validation. The calculated methylation levels are shown in the Fig 9. We found that the methylation level of all the 5 regions agreed with MBD-Seq analysis. The bisulfite sequencing employed only a segment of the methylation region to perform the validation, thus the magnitude of methylation difference of the validation results could not be exactly the same as MBD-Seq analysis.

For the differentially expressed miRNA bta-mir-122, the expression level in grain-fed ruminal wall was significantly higher than in grass-fed ruminal wall, and the results of qPCR and RNA-Seq suggested the same direction (S2 Fig).

### Expression profiles of microRNAs in grass-fed and grain-fed groups

**Fig 10:**
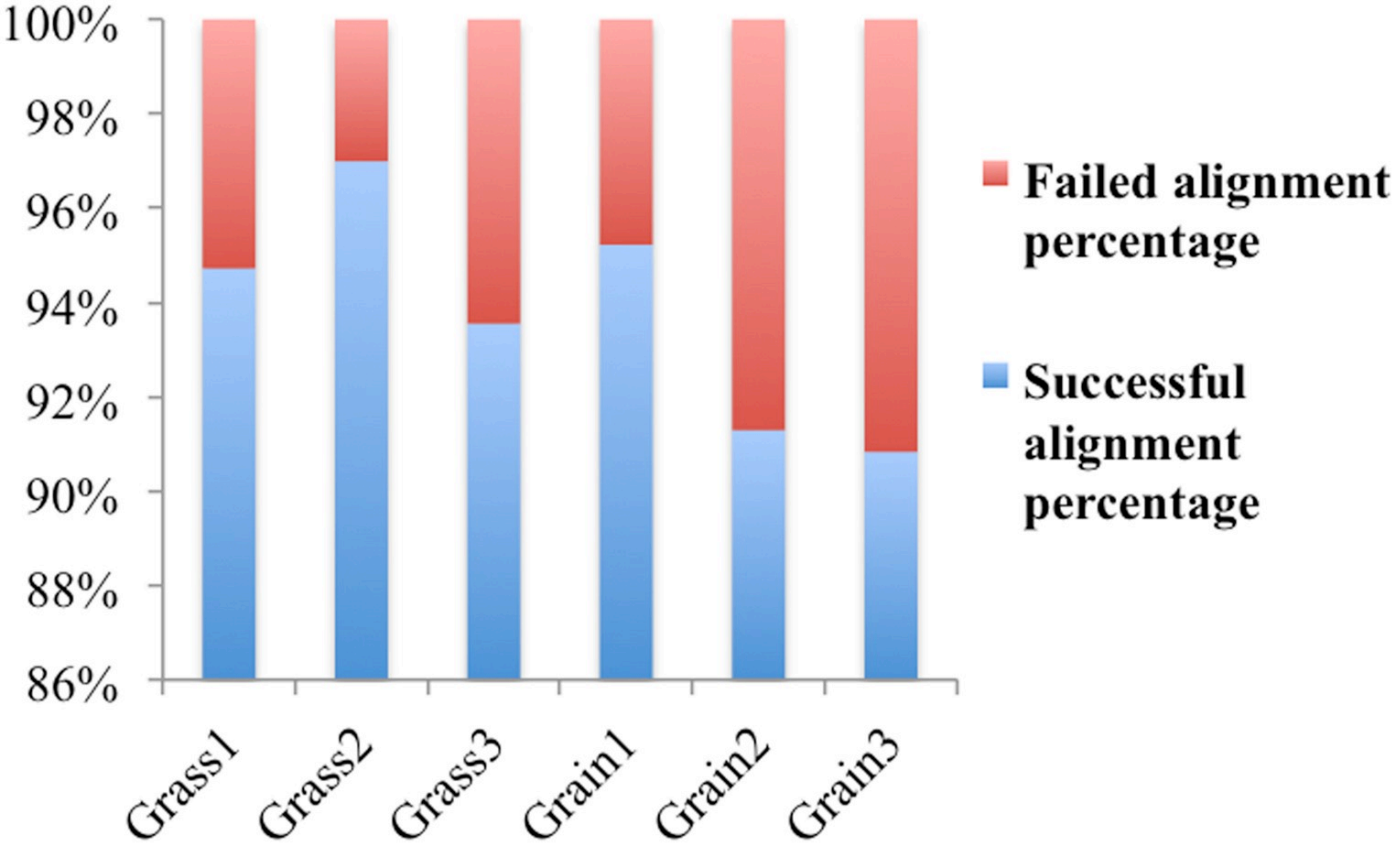
Alignment level of miRNA-Seq reads to the bovine genome.

To identify the microRNAs involved in bovine rumen function, total RNAs from three biological replicates for each condition were used to construct small RNA libraries. Via high-throughput sequencing, we obtained an average of 15 million reads per sample. The percentage of alignment for all samples exceeded 90% as shown in Fig 10. Based on the aligned sequencing reads, we totally identified 321 known miRNAs (S7 Table), of which 72.9% were high expressed in grain-fed individuals compared with grass-feed ones. Between the two groups, we identified only one differentially expressed miRNA (FDR < 0.1), which was bta-mir-122 with improved expression in grain-fed group as compared with grass-fed animas. Additionally, we found that microRNA bta-mir-655 was exclusively expressed in the grain-fed group.

### Target gene prediction, Gene Ontology and pathways enriched by the differentially expressed microRNAs

Prediction of target genes of the miRNA was performed using TargetScan (http://www.targetscan.org/). For bta-mir-122 and bta-mir-655, we respectively found 145 and 749 target genes. To explore the specific functional features shared by the targets of the above miRNA, online software David Bioinformatics Resources 6.7 was used to perform the GO enrichment analysis, mainly regarding the biological processes, cellular components and molecular functions. For the targets of bta-mir-122, the most significant GO terms were: regulation of GTPase activity, regulation of protein signal transduction, intracellular, GTPase activator activity and GTPase regulator activity (Table 1). To some extent, the targets were also involved in the pathway glycolysis/gluconeogenesis (P = 7.9×10^−2^), which could produce energy and is an critical metabolic pathway employed by a host of organisms. Therefore, this might be an interesting pathway to study in rumen. For the targets of bta-mir-655, the most significant GO terms of the three categories were: regulation of cellular process, regulation of biological process, biological regulation (Table 2). The complete list of GO terms enriched with the target genes of bta-mir-655 was provided in S8 Table. Then, we detected the pathways that involved the targets of bta-mir-655, the most significantly enriched pathways were shown in Table 3. Several interesting pathways were found including adherens junction, insulin signaling pathway, TGF-beta signaling pathway and neurotrophin signaling. These results would provide prior knowledge to explain the difference between grass-fed and grain-feed Angus cattle.

**Table 1:** Gene Ontology (GO) terms enriched with the targets of bta-mir-122 (P < 0.01).

GO terms	Observed*	P
***Biological process***		
GO:0032318~regulation of Ras GTPase activity	5	1.00×10^−3^
GO:0043087~regulation of GTPase activity	5	1.78×10^−3^
GO:0032313~regulation of Rab GTPase activity	4	2.27×10^−3^
GO:0032483~regulation of Rab protein signal transduction	4	2.27×10^−3^
GO:0046578~regulation of Ras protein signal transduction	6	3.81×10^−3^
***Cellular component***		
GO:0005622~intracellular	53	7.16×10^−3^
***Molecular function***		
GO:0005097~Rab GTPase activator activity	4	1.97×10^−3^
GO:0005099~Ras GTPase activator activity	4	4.51×10^−3^
GO:0005083~small GTPase regulator activity	6	5.07×10^−3^
GO:0005096~GTPase activator activity	5	5.68×10^−3^

*Number of the differentially expressed genes in the category

**Table 2:** The top Gene Ontology (GO) terms enriched with the targets of bta-mir-655 (P < 0.01).

GO terms	Observed*	P
***Biological process***		
GO:0050794~regulation of cellular process	152	8.72×10^−14^
GO:0050789~regulation of biological process	156	6.32×10^−13^
GO:0065007~biological regulation	160	1.35×10^−11^
GO:0007275~multicellular organismal development	68	6.40×10^−10^
GO:0032502~developmental process	72	1.27×10^−9^
GO:0051171~regulation of nitrogen compound metabolic process	74	1.91×10^−8^
GO:0019222~regulation of metabolic process	87	2.15×10^−8^
GO:0031326~regulation of cellular biosynthetic process	76	2.22×10^−8^
GO:0009889~regulation of biosynthetic process	76	2.72×10^−8^
GO:0019219~regulation of nucleobase, nucleoside, nucleotide and nucleic acid metabolic process	73	3.25×10^−8^
***Cellular component***		
GO:0005622~intracellular	231	5.75×10^−9^
GO:0044424~intracellular part	204	7.65×10^−7^
GO:0043231~intracellular membrane-bounded organelle	156	1.46×10^−5^
GO:0043227~membrane-bounded organelle	156	1.64×10^−5^
GO:0005634~nucleus	101	2.88×10^−5^
GO:0043229~intracellular organelle	170	1.15×10^−4^
GO:0043226~organelle	170	1.21×10^−4^
GO:0044451~nucleoplasm part	18	7.37×10^−4^
GO:0005654~nucleoplasm	19	1.80×10^−3^
GO:0005938~cell cortex	9	2.06×10^−3^
***Molecular function***		
GO:0005488~binding	314	2.95×10^−11^
GO:0030528~transcription regulator activity	50	5.26×10^−7^
GO:0005515~protein binding	176	1.95×10^−6^
GO:0043565~sequence-specific DNA binding	27	1.44×10^−5^
GO:0003700~transcription factor activity	33	3.45×10^−5^
GO:0008134~transcription factor binding	14	2.52×10^−4^
GO:0008270~zinc ion binding	68	3.61×10^−4^
GO:0046914~transition metal ion binding	74	6.84×10^−3^
GO:0003677~DNA binding	49	8.13×10^−3^
GO:0016563~transcription activator activity	10	9.18×10^−3^

*Number of the differentially expressed genes in the category

**Table 3:** KEGG pathways enriched with the target genes of bta-mir-655 by GO analysis (P < 0.01).

KEGG Pathway	Observed*	P value
Adherens junction	10	2.32×10^−4^
Insulin signaling pathway	13	5.49×10^−4^
TGF-beta signaling pathway	9	3.35×10^−3^
Neurotrophin signaling pathway	11	4.03×10^−3^
Renal cell carcinoma	8	4.24×10^−3^
Pancreatic cancer	8	5.03×10^−3^
Fc gamma R-mediated phagocytosis	9	5.64×10^−3^
Pathways in cancer	19	7.07×10^−3^
Axon guidance	10	9.87×10^−3^

*Number of the target genes in the pathway

### Combined analysis of microRNA-Seq with transcriptome (RNA-Seq)

For bta-mir-122, two of the 145 target genes were found in the DEGs list from RNA-Seq, which were OCLN and RBM47, respectively. These two genes were all highly expressed in grass-fed group, showing a negative correlation with bta-mir-122, while the difference was not significant for the correlation (p > 0.05). For bta-mir-655 exclusively expressed in grain-fed animals, we found that 14 of the 749 targets were overlapped with the DEGs. Among the 14 overlapped genes, four genes displayed increased expression in grain-fed group, among which GAS1, ANO1 and NFASC displayed significantly positive correlation with bta-mir-655 (p < 0.05); the other nine genes (MYO10, SASH1, EMP1, SLC14A1, PCDH19, IRX5, MAL2, FAM84A, AHCYL2 and DSG3) exhibited enhanced expression level in grass-fed group, which demonstrated negative correlation with bta-mir-655, but none of them showed significant difference (p > 0.05) (S9 Table).

## Discussion

In addition to genetic makeup, age, species, gender and environment, grass and grain ratio of the diet could also contribute to significant difference in the general profile of fatty acid, animals’ growth rate, the content of vitamin A and E in the meat, the antioxidant enzyme concentration in body tissues and lipid depots [10, 40–41]. In the past several years, miRNA and DNA methylation have been studied widely. However, limited studies have focused on the rumen tissue of bovine, one of the most significant livestock in the world. Rumen was the most important workshop for the digestion of nutritional substance of bovine. The potential changes of rumen metabolism may have effects on the quality and quantity of protein, affecting other digestive organs, such as reticulum, small intestine and large intestine. Therefore, rumen function was critical for animals’ growth, health and productivity. In the present study, we mainly focused on the rumen under different feeding diets, detecting the genome-wide DNA methylation profiles and miRNA expression profiles, and we also performed combined analysis of these with transcriptomic results. The objective was to identify the DMRs and miRNAs that might potentially affect the rumen function. This study performed a comprehensive analysis of DNA methylation profiles in the rumen tissues of grass-fed and grain-fed animals, and totally revealed 217 DMRs across the whole genome. In our analysis, we found that 21 DMRs were located in the promoter regions; however, none of their corresponding genes were overlapped with the DEGs. For these DMRs, although the methylation level was different, the concentration might not be high enough to cause the expression difference of their targets. Or, these DMRs could actually exert no effects on the genes. Additionally, we detected 57 DMRs inside 52 genes, of which two genes (ADAMTS3 and ENPP3) were overlapped with the DEGs. The methylation abundance of the DMRs exhibited negative and positive correlation with the expression of the corresponding gene ADAMTS3 and ENPP3, respectively. Studies suggested that DNA methylation in the gene body region might change the chromatin structure and alter the transcription elongation efficiency [18]. Gene body methylation was more prevalent than promoter; its relationship with gene expression levels was very complex and not monotonic. Normally, gene body DNA methylation showed positive correlation with gene expression [42]. However, the information about the role of DNA methylation in the gene body region was still insufficient. For the two DEGs predicted to be correlated with the DMR in the gene body region, ADAMTS3 belonged to the ADAMTS (a disintegrin and metalloproteinase domain with thrombospondin motifs) metalloproteinase family mediating cartilage aggrecan degradation as well as collagen biosynthesis [43]. It was involved in the biosynthesis of type II procollagen, the main collagen of articular cartilage [44]. ENPP3 was known to be a typical ectoenzyme localized to the cell surface, playing a role in metabolizing extracellular nucleotides and their derivatives [45]. And, it was suggested that ENPP3 could modulate nucleotide-mediated signal transduction, which was known as purinergic signaling [46–47]. Recent study demonstrated that ENPP3 could catalyze the hydrolysis of the nucleotide sugar, and the levels of several intracellular sugars, including UDP-Fuc, UDP-GalNAc and UDP-GlcA, were significantly influenced by knocking down endogenous ENPP3 [48]. There was evidence showing that intracellular sugars played a role in regulating glycosyltransferase activity and controlling the total cellular glycosylation profile [49–51]. Therefore, through DNA methylation regulation, the expression of ENPP3 would be changed, which might indirectly regulate the activity of a broad range of glycosyltransferase and consequently influence the total cellular glycosylation pattern.

The miRNAs could negatively regulate gene expression through degrading the target mRNAs or inhibiting the translation [52]. However, the understanding of the contribution of miRNA to rumen function was still limited. In this study, we aimed to get a deeper insight into underlying mechanism of miRNA in rumen function. Between grass-fed and grain-fed group, we found one differentially expressed miRNA (bta-mir-122) with higher expression level in grain-fed steers. Bta-mir-122 was a conserved miRNA between vertebrate species. It was also found differentially expressed between normal and aberrant placental samples, suggesting that bta-mir-122 might be involved in the development of placentae [53]. In liver, bta-mir-122 was relevant in maintenance of homeostasis and had critical metabolic and anti-inflammatory functions [54]. In our study, we detected 145 target genes for bta-mir-122 in total. To explore the specific functional features shared by the 145 targets, we performed GO enrichment analysis. Results showed that the targets were mainly involved in the GTPase activity and regulation of protein signal transduction. GTP hydrolysis played an essential role in controlling numerous biological processes, including protein biosynthesis, growth control and differentiation, and various transport processes. Additionally, two of the 145 target genes were found in the DEGs list, which were OCLN and RBM47, respectively. OCLN was a key tight junction protein that could interact with intracellular signaling pathways, which played roles in regulating intestinal function [55]. RBM47 was a novel RNA-binding protein; it could contribute to the basic machinery for C to U RNA editing in intestine, influencing the expression of some genes [56]; however, the function of this gene has not been studied in rumen. We made the hypothesis that RBM47 would alter the expression of some genes in rumen, consequently regulating rumen function. Meanwhile, we detected one exclusively expressed miRNA (bta-mir-655) in grain-fed group; it could target 749 different genes. After the GO analysis based on the targets, the most significant GO terms included regulation of cellular process, biological regulation, developmental process, regulation of metabolic process, and transcription factor activity, which played a critical role in rumen function. The most interesting pathways included adherens junction, insulin signaling pathway and TGF-beta signaling pathway, which were related to animals’ growth, survival and development. In addition, 14 of the 749 targets were overlapped with the DEGs; however, none of them was discovered in the pathways. Thus, we hypothesized that bta-mir-655 just slightly changed the expression of the targets without significant expression change; but the target genes working together in the corresponding pathway could exert significant effects on the rumen function. Among the 14 overlapped genes, FAM84A, localized in the subcellular membrane region, was involved in invasion and/or metastasis of colon cancer cells influencing colorectal cancer [57]; SASH1 was a member of the SH3-domain containing expressed in lymphocytes (SLY1) gene family, it encodes signal adapter proteins which were composed of certain protein–protein interaction domains, showing prognostic significance in human cancer [58]. A previous study suggested that epithelial membrane protein 1 (EMP1) gene could prevent tumor proliferation and was associated with gastric carcinoma [59]. DSG3 was predicted to be involved in the GO term cell adhesion; it was reported to be associated with oncogenesis [60]. However, information about the function of these genes in rumen was still limited; they might play a potential role in animal development through affecting the gastrointestinal function. Accordingly, it might be of great interest to perform functional experiment of these genes to better understand the mechanisms causing the varied performance.

Our results provided evidence for explaining the molecular mechanisms leading to the differences between grass-fed and grain-fed cattle. For the function of the DEGs containing the DMR and the differentially expressed miRNAs, extensive experimental validation work was still needed. Thus, overexpression and inhibition of our identified DEGs and miRNAs could be considered for the functional validation, which would provide more supportive information for our findings.

## Conclusions

In this study, we combined DNA methylation and miRNA expression with transcriptome analysis to explore the potential mechanism influencing rumen function of grass-fed and grain-fed animals. We found that the expression of ADAMTS3 and ENPP3 might be altered by the corresponding DMR inside these two genes, through which rumen function of grass-fed and grain-fed cattle could be regulated. For the differentially expressed miRNA bta-mir-122, it might modulate the rumen function by targeting the two DEGs OCLN and RBM47, which were possibly associated with gastrointestinal function. While expanding the scope of future studies with putative genes relevant to bovine growth and meat quality traits, our analysis provided biological insights into the mechanisms regulating rumen function and uncovered the molecular basis underlying the economic traits to enhance the productivity of animals.

## Supporting information

S1 Fig Alignment level of MBD-Seq reads to the bovine genome. (TIF)

S2 Fig Validation of differentially expressed miRNA. The mean value of log2 (fold-change) for each group was compared in the bar chart. FC means fold-change.
(TIF)

S1 Table Primers used for validating the randomly selected DMRs. (XLS)

S2 Table Primers used for validating the differentially expressed miRNA. (XLS)

S3 Table The DMRs between grass-fed and grain-fed Angus cattle. The threshold of FDR <0.1 was used to call the significant difference.
(XLS)

S4 Table Annotation of the identified DMRs. (XLS)

S5 Table The association analysis of the identified DMRs located within the genes promoters and their corresponding genes. (XLSX)

S6 Table The association analysis of the identified DMRs inside the genes and their corresponding genes. (XLSX)

S7 Table The known miRNAs in grass-fed and grain-fed animals. (XLSX)

S8 Table The GO terms enriched with the target genes of bta-mir-655. (XLS)

S9 Table The association analysis between bta-mir-122 and bta-mir-655 and their targeted DEGs. (XLSX)
