# Supplementary figures and images for: DNA methylation, microRNA expression profiles and their relationships with transcriptome in grass-fed and grain-fed Angus cattle rumen tissue
Source: PLoS One. 2019 Oct 17;14(10):e0214559. doi: 10.1371/journal.pone.0214559 (PMC6797229; doi:10.1371/journal.pone.0214559)

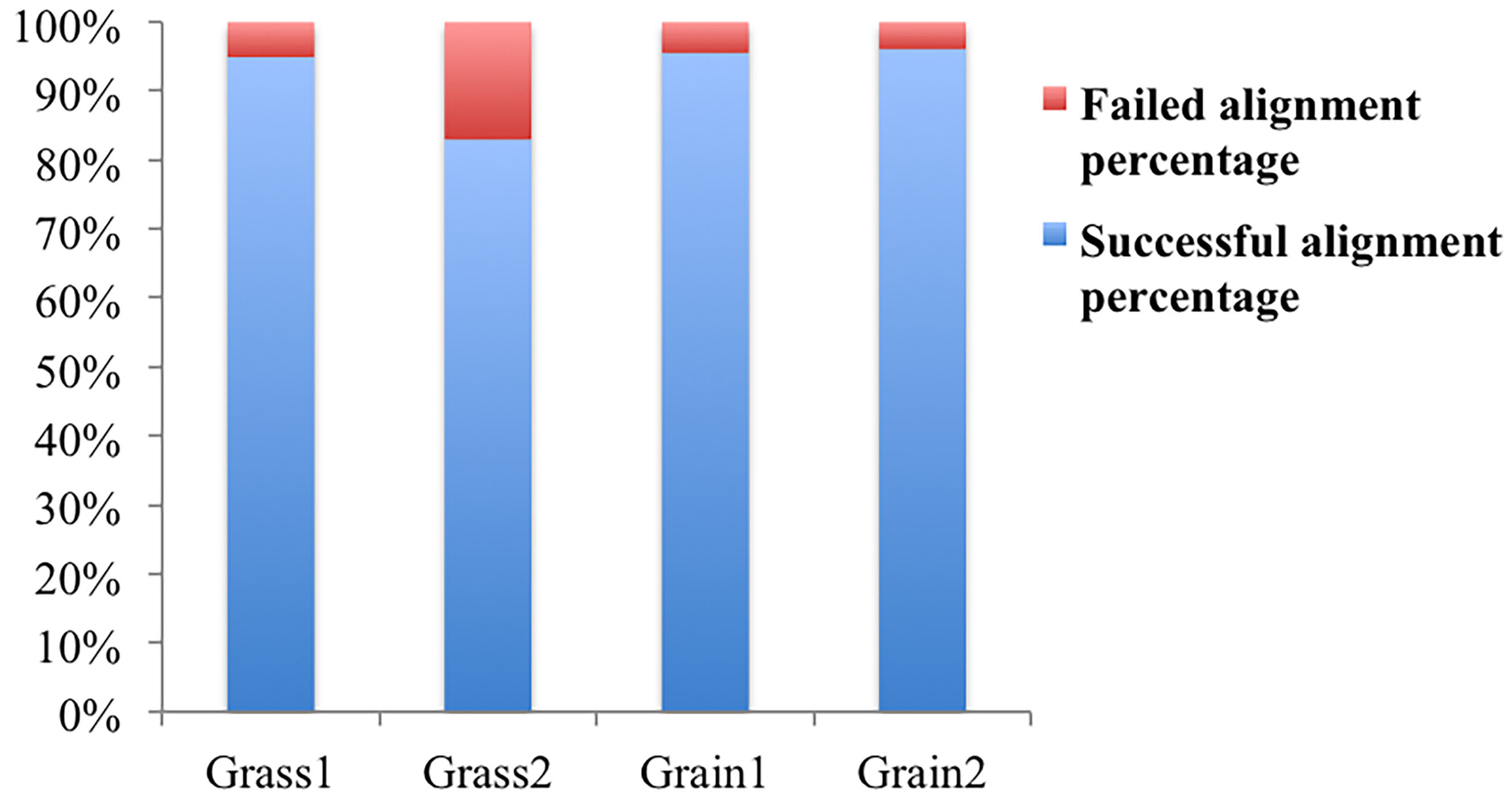

Supplement: S1 Fig — (TIF) [file pone.0214559.s001.tif]

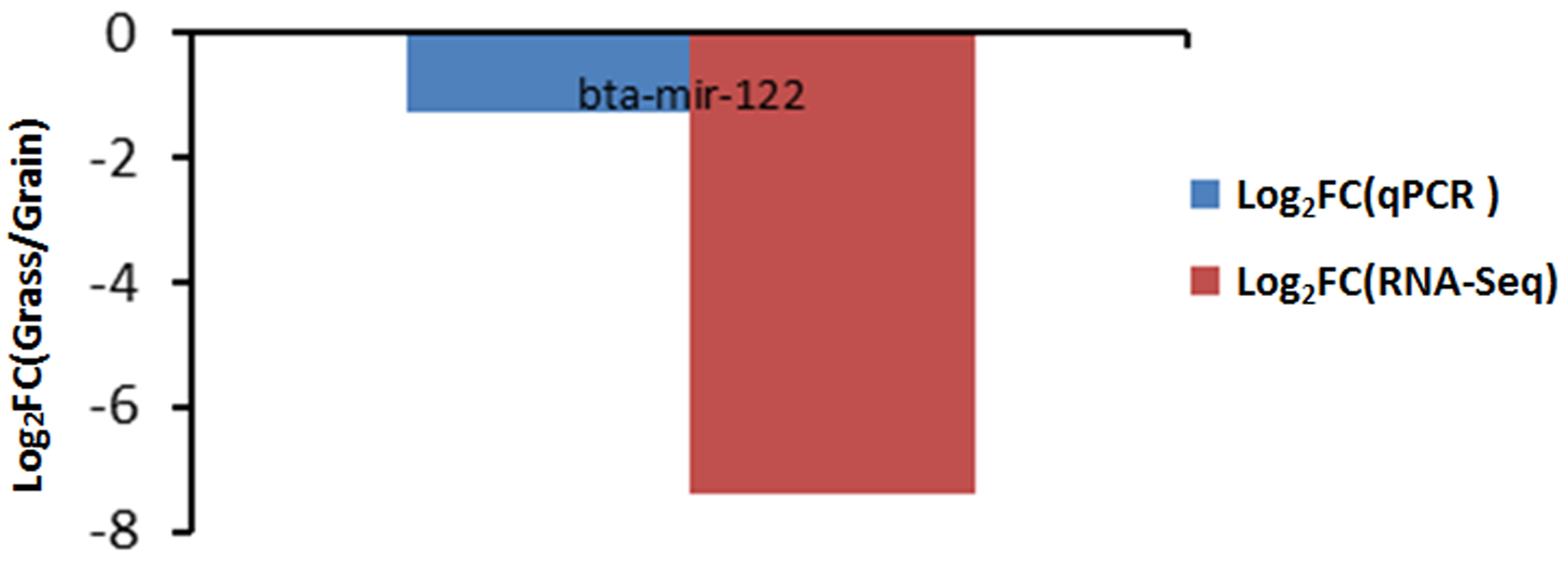

Supplement: S2 Fig — The mean value of log2 (fold-change) for each group was compared in the bar chart. FC means fold-change. (TIF) [file pone.0214559.s002.tif]
